# In Silico and In Vitro Study of Janus Kinases Inhibitors from Naphthoquinones

**DOI:** 10.3390/molecules28020597

**Published:** 2023-01-06

**Authors:** Kamonpan Sanachai, Panupong Mahalapbutr, Lueacha Tabtimmai, Supaphorn Seetaha, Nantawat Kaekratoke, Supakarn Chamni, Syed Sikander Azam, Kiattawee Choowongkomon, Thanyada Rungrotmongkol

**Affiliations:** 1 Center of Excellence in Biocatalyst and Sustainable Biotechnology, Department of Biochemistry, Faculty of Science, Chulalongkorn University, Bangkok 10330, Thailand; 2 Department of Biochemistry, Center for Translational Medicine, Faculty of Medicine, Khon Kaen University, Khon Kaen 40002, Thailand; 3 Department of Biotechnology, Faculty of Applied Science, King Mongkut’s University of Technology of North Bangkok, Bangkok 10800, Thailand; 4 Department of Biochemistry, Faculty of Science, Kasetsart University, Bangkok 10900, Thailand; 5 Department of Materials Science and Engineering, Vidyasirimedhi Institute of Science and Technology (VISTEC), Rayong 21210, Thailand; 6 Department of Pharmacognosy and Pharmaceutical Botany, Faculty of Pharmaceutical Sciences, Chulalongkorn University, Bangkok 10330, Thailand; 7 Natural products and Nanoparticles Research Unit (NP2), Faculty of Pharmaceutical Sciences, Chulalongkorn University, Bangkok 10330, Thailand; 8 Computational Biology Lab, National Center for Bioinformatics, Quaid-i-Azam University, Islamabad 45320, Pakistan; 9 Program in Bioinformatics and Computational Biology, Graduate School, Chulalongkorn University, Bangkok 10330, Thailand

**Keywords:** JAK2/3 inhibitors, naphthoquinones, enzymatic and cell-based assay, molecular dynamics simulations

## Abstract

Janus kinases (JAKs) are involved in numerous cellular signaling processes related to immune cell functions. JAK2 and JAK3 are associated with the pathogenesis of leukemia and common lymphoid-derived illnesses. JAK2/3 inhibitors could reduce the risk of various diseases by targeting this pathway. Herein, the naphthoquinones were experimentally and theoretically investigated to identify novel JAK2/3 inhibitors. Napabucasin and 2′-methyl napabucasin exhibited potent cell growth inhibition in TF1 (IC_50_ = 9.57 and 18.10 μM) and HEL (IC_50_ = 3.31 and 6.65 μM) erythroleukemia cell lines, and they significantly inhibited JAK2/3 kinase activity (in a nanomolar range) better than the known JAK inhibitor, tofacitinib. Flow cytometric analysis revealed that these two compounds induced apoptosis in TF1 cells in a time and dose-dependent manner. From the molecular dynamics study, both compounds formed hydrogen bonds with Y931 and L932 residues and hydrophobically contacted with the conserved hinge region, G loop, and catalytic loop of the JAK2. Our obtained results suggested that napabucasin and its methylated analog were potential candidates for further development of novel anticancer drug targeting JAKs.

## 1. Introduction

Janus kinases (JAKs) in the enzyme family of tyrosine kinases are known for their ability to phosphorylate tyrosine residues, changing the function of the protein in which they are present. They can transfer extracellular signals from cell surface receptors to the nucleus, affecting DNA transcription and the subsequent translation of proteins. The JAK-STAT pathway, one of the immune system communication pathways, functions downstream of more than 50 cytokines and growth factors [1]. The JAK family contains four members: JAK1, JAK2, JAK3, and TYK2. Each cell surface receptor requires a pair of JAKs that are either identical homodimers or heterodimers [2]. Signal transducers and activators of transcription (STAT) proteins are then activated, targeting gene promoters to start transcription. The activating ligands and subsequent effector functions of any pair of JAKs are unique [3]. Among the JAK members, JAK2 participates in multiple cytokine pathways, but its contribution to IL6-mediated signaling is crucial for several physiological processes, including B-cell differentiation, hematopoiesis, and bone metabolism. An imbalance in IL-6-mediated signaling can promote B-cell differentiation, which further contributes to illnesses with lymphoid-derived diseases [4]. JAK3 controls the activity of many lymphoid cell types, including T and B lymphocytes and natural killer (NK) cells, and so it is essential for lymphoid development [2]. The pathogenesis of illnesses with lymphoid-derived disease is also influenced by the imbalance in IL-2-mediated signaling [5]. Therefore, the development of dual JAK2/3 inhibitors may be an effective way to treat a range of illnesses arising from lymphoid cells that rely on the JAK2 and JAK3 signaling cascade.

In clinical trials, the dual JAK2/3 inhibitor AG-490 significantly prevents B-cell growth in overcontrol in patients with acute lymphoblastic leukemia by preventing the inconsistent constitutive activation of JAK2 [6]. In addition, there are currently two JAK inhibitors on the market: the first is ruxolitinib, a JAK1 (IC_50_ = 0.09 nM)/JAK2 (IC_50_ = 0.036 nM) inhibitor that was licensed in late 2011 for the treatment of myelofibrosis [7,8]. Tofacitinib (IC_50_ = 1.7–3.7 nM for JAK1, 1.8–4.1 nM for JAK2, and 0.75–1.6 nM for JAK3) was authorized to treat rheumatoid arthritis a year later [9,10,11,12,13]. Naphthoquinones are considerably favored structures in medicinal chemistry due to their biological activity and structural characteristics. A wide range of biological activities of naphthoquinone-based compounds, including anti-inflammatory, cytotoxic, and anticancer effects, have been reported [14]. Some quinone-based antitumor agents have been used to treat solid tumors (e.g., doxorubicin) [15] or acute lymphoblastic and myeloblastic leukemias (e.g., daunorubicin) [16]. JAK inhibitors derived from naphthoquinone derivatives have been reported [17,18,19]. Three Chinese herbal compounds, cryptotanshinone (25 μM), icaritin (20 μM), and indirubin (50 μM), exhibited JAK3 inhibition from the enzymatic assay. Furthermore, the finding provides binding models of the protein–ligand interactions that detail the interacting amino acid residues in the functional ATP-binding domains of JAK3 kinase. For example, cryptotanshinone, icaritin, and indirubin interact with V836, A853, Y904, and L956 by van der Waals interaction, while hydrogen bonds formations with E903, L905, A966, and D967 were found only in icaritin binding [17]. Lin et al. reported that the naphtho [1,2-b]furan-4,5-dione disrupts JAK2 and induces apoptosis in human breast cancer MDA-MB-231 cells [18]. In addition, 2-methoxystypandrone, derived from the traditional Chinese medicinal herb *Polygonum cuspidatum,* has been reported to inhibit JAK2 in both human adenocarcinoma and carcinoma [19]. Furthermore, various naphthoquinones have been widely reported to exhibit anticancer effects. For example, the results of experiments and 3D-QSAR study suggest that naphtho [2,3-b]thiophene-4,9-dione analogs are promising in the development of new anti-colorectal cancer agents [20]. In vitro assays proved that the hybrid molecule of three component reaction of 2-hydroxy-1,4-naphthoquinone (lawsone), 4-hydroxycoumarin, and 3,4-dimethoxybenzaldehyde under solvent-free conditions could inhibit chronic myelogenous leukemia cell proliferation with the IC_50_ value in a micromolar range [21]. 

**Figure 1:**
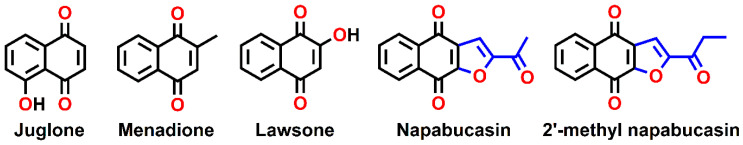
Two-dimensional structure of naphthoquinone analogs.

A series of naphthoquinones, i.e., juglone, menadione, lawsone, napabucasin, and 2′-methyl napabucasin, were selected based on their 1,4-naphthoquinone core with hydroxyl, methyl, and furanoyl substitutions (Figure 1). Juglone and menadione were isolated from plants in the Juglandaceae family, particularly the black walnut (*Juglans nigra*) and English walnut (*Juglans regia*) [22]. Lawsone was obtained from the leaves of *Lawsonia inermis* [23]. Napabucasin is a plant-produced furanonaphthoquinone found in the Bignoniaceae family, including *Ekmanianthe longiflora* [24] and *Tabebuia avellanedae* [25]. 2′-Methyl napabucasin is a synthetic furanonaphthoquinone analog that exhibited potent cytotoxicity against human keratinocytes [26,27]. In addition, napabucacin has been continuously considered as an anticancer agent in various targets, such as Akt/mTOR [28] and EGFR [29] involved in lung cancer and STAT3 associated with solid tumors [30,31,32]. However, its ability in JAK inhibitions has not yet been reported. Therefore, we aimed to investigate the JAK2/3 inhibitions of these five naphthoquinones by experimental and theoretical techniques. The naphthoquinones’ cytotoxicity and ability to inhibit kinases were evaluated in human erythroleukemia cell lines expressing JAK2, TF1 (WT), and HEL (V617F). The dose- and time-dependent apoptosis was determined using flow cytometric analysis. Finally, molecular dynamics simulations and free energy calculations were used to elucidate the molecular interactions of the potent compounds with JAK2.

## 2. Results and Discussion 

### 2.1. Cytotoxicity on Leukemia Cell Lines

**Figure 2:**
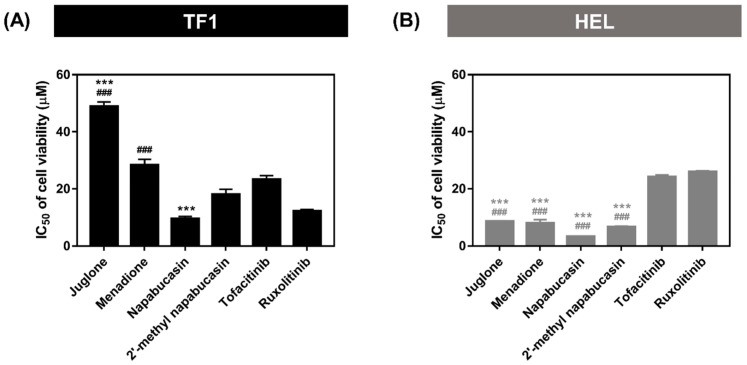
The IC_50_ of naphthoquinones against (**A**) TF1 and (**B**) HEL cell viability by Presto Blue assay. *** *p* ≤ 0.001 and ^###^ *p* ≤ 0.001 compared with tofacitinib and ruxolitinib, respectively.

Five naphthoquinones at 10 μM were screened for their cytotoxicity on leukemia wild-type TF1 and mutant HEL cells overexpressing JAK2 by Presto Blue assay. Commercial drugs, tofacitinib and ruxolitinib, were used as the positive control (Figure S1). The napabucasin and 2′-methyl napabucasin revealed highly potent cell growth inhibition towards both TF1 and HEL cells, comparable with both drugs. Juglone and menadione showed high cytotoxicity only in HEL cells. Four naphthoquinones, napabucasin, 2′-methyl napabucasin, juglone, and menadione, exhibited effective inhibition either of TF1 or HEL cells. Lawsone inhibited the growth of both cell types poorly. Therefore, four compounds, except lawsone, were evaluated for IC_50_ towards both cells (Figure 2, Figures S2 and S3). 

For TF1 cells, 2′-methyl napabucasin (IC_50_ 18.10 ± 1.71 μM) inhibited the cell growth at a similar level to tofacitinib (IC_50_ 23.30 ± 1.37 μM) and ruxolitinib (IC_50_ 12.24 ± 0.58 μM). Napabucasin (IC_50_ 9.57 ± 0.79 μM) showed an effective inhibition toward TF1 cells better than tofacitinib. In addition, HEL cells treatment with juglone (IC_50_ 8.67 ± 0.17 μM) and menadione (IC_50_ 7.99 ± 1.22 μM) gave potent cytotoxic activity greater than the drugs tofacitinib (IC_50_ 24.19 ± 0.67 μM) and ruxolitinib (IC_50_ 26.04 ± 0.26 μM) by about three times. Furthermore, the furanonaphthoquinone series, including napabucasin (IC_50_ 3.31 ± 0.06 μM) and 2′-methyl napabucasin (6.65 ± 0.29 μM), displayed a cytotoxic activity toward HEL cells higher than both drugs, about six and four-fold for tofacitinib and ruxolitinib, respectively. In a previous report, naphtoquinone-coumarin conjugate (NPQC6; IC_50_ = 1.30 μM) [33] and naphtoquinone-benzonitrile conjugate (CM363; IC_50_ = 1.30 μM) [14] can inhibit HEL cell growths at a similar level of napabucasin and 2′-methyl napabucasin in this work. Furthermore, the NPQC6 also displayed myelogenous leukemia K562 (IC_50_ = 1.40 μM) and HL60 (IC_50_ = 0.30 μM) cell growth inhibitions [33]. Our IC_50_ values demonstrated that four compounds, juglone, menadione, napabucasin, and 2′-methyl napabucasin, inhibited mutant HEL cells better than wild-type TF1. This is because the V671F mutation maintains an activation loop for open conformation, resulting in a higher binding ability of the ligand [34,35]. The four compounds, juglone, menadione, and two napabucasins, were then selected to perform the kinase inhibition assay. 

### 2.2. Kinase Inhibitory Activity of JAK2/3

**Figure 3:**
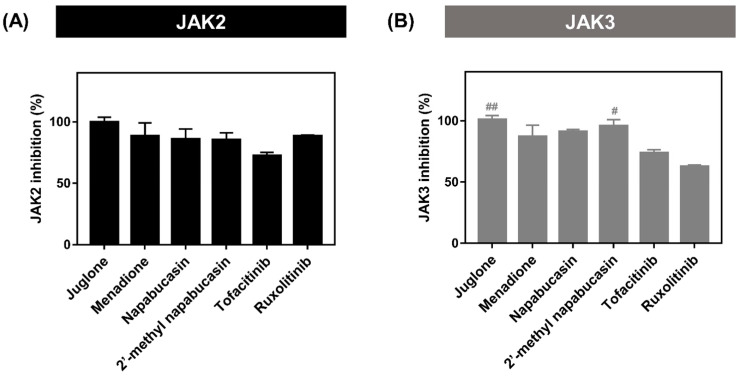
(**A**) JAK2 and (**B**) JAK3 inhibitory activity of naphthoquinones at 1 μM by ADP-Glo™ kinase assay. ^#^ *p* ≤ 0.05 and ^##^ *p* ≤ 0.01 compared with ruxolitinib.

The kinase inhibitory activities towards JAK2/3 of four naphthoquinones (juglone, menadione, napabucasin, and 2′-methyl napabucasin), which significantly showed TF1 or HEL cell growth inhibitions, were screened and compared with tofacitinib and ruxolitinib at 1 μM by ADP-Glo™ kinase assay (Figure 3). The result revealed that all compounds gave JAK2 and JAK3 inhibition by more than 80%. However, napabucasin and 2′-methyl napabucasin were selected for IC_50_ analysis due to their highly potent inhibition of TF1 and HEL cell growth (Figure 2). The IC_50_ value of the napabucasin series was summarized in Table 1 and Figure S4. It was found that both compounds, napabucasin (IC_50_ = 12.62 ± 2.12 nM) and 2′-methyl napabucasin (11.11 ± 0.13 nM), potently inhibited JAK2 higher than tofacitinib (32.10 ± 1.99 nM), by about three-fold, while they presented the inhibition at a similar level with ruxolitinib (14.63 ± 0.81 nM). Both napabucasins resulted in JAK2 inhibition, similar to the previous report showing that the 2,8-diaryl-quinoxalines consisting of 4-phenylacetic morpholine amide can inhibit JAK2 activity with an IC_50_ value of 13 nM [36]. For JAK3 inhibition, 2′-methyl napabucasin (10.24 ± 0.66 nM) exhibited a greater inhibitory than tofacitinib (21.03 ± 0.97 nM) by about two-fold. However, no significant difference (*p* > 0.05) in JAK3 inhibition was observed between napabucasin (14.84 ± 2.37 nM) and tofacitinib. Altogether, it can be concluded that napabucasin and 2′-methyl napabucasin can inhibit both JAKs; thus, both compounds were selected to further studies. 

### 2.3. Apoptosis on TF1 Cell Treated with Napabucasin

**Figure 4:**
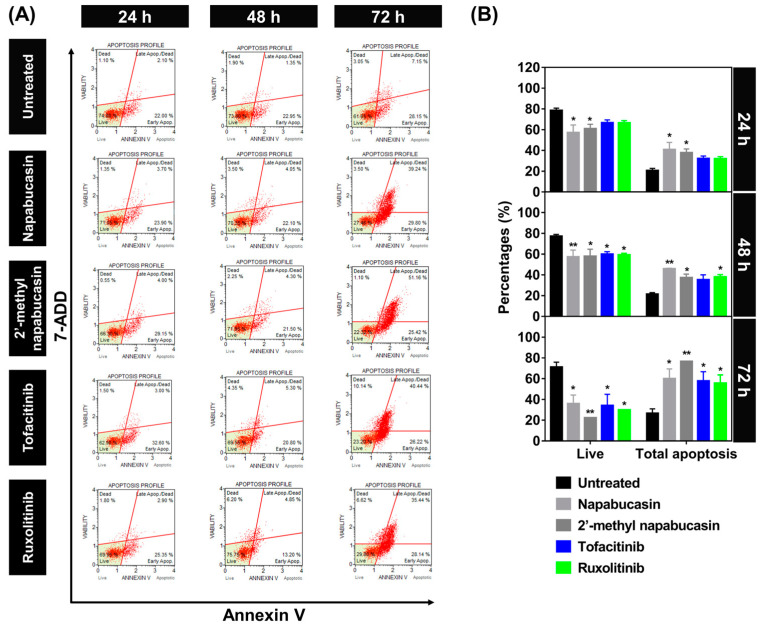
Time-dependent effect of napabucasin and 2′-methyl napabucasin comparison with drugs, tofacitinib, and ruxolitinib at IC_50_ values on the apoptosis of TF1 cells by annexin V (24, 48, and 72 h). (**A**) The flow cytometry dot plot of each group is divided into four quadrants: live cells, early apoptotic cells, late apoptotic cells, and necrotic cells. (**B**) The percentages of live cells and apoptotic cells. * *p* ≤ 0.05, ** *p* ≤ 0.01 and *** *p* ≤ 0.001 compared with the untreated group.

**Figure 5:**
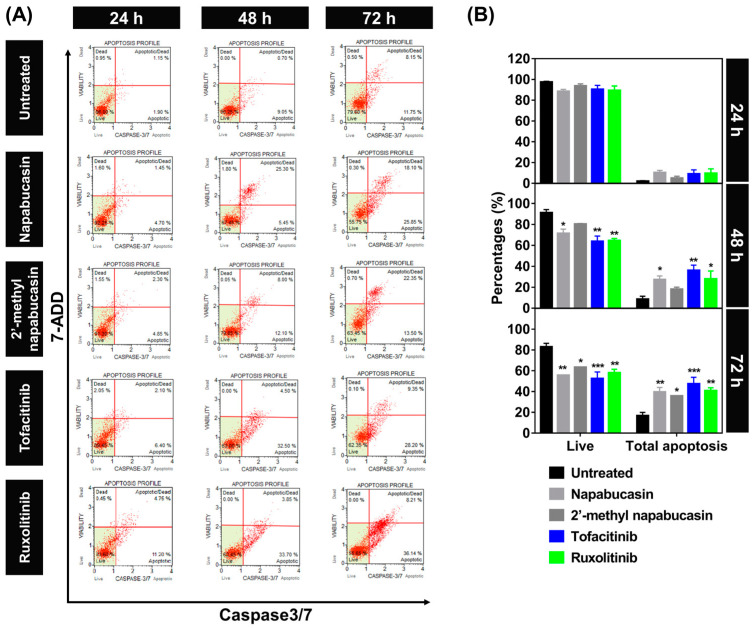
Time-dependent effect of napabucasin and 2′-methyl napabucasin comparison with drugs tofacitinib and ruxolitinib at IC_50_ values on the apoptosis of TF1 cells by caspase 3/7 (24, 48, and 72 h). (**A**) The flow cytometry dot plot of each group is divided into four quadrants: live cells, early apoptotic cells, late apoptotic cells, and necrotic cells. (**B**) The percentages of live cells and apoptotic cells. * *p* ≤ 0.05, ** *p* ≤ 0.01 and *** *p* ≤ 0.001 compared with the untreated group.

The apoptosis-inducing effect of napabucasin and 2′-methyl napabucasin on TF1 cell death was analyzed by annexin V (Figure 4) and caspase 3/7 (Figure 5) detection at various concentrations for 24, 48, and 72 h, respectively, which was measured by flow cytometric analysis. The human erythroleukemia TF-1 cell was used as a model because this cell expresses wild-type JAK2. Among the group of 24-h treatment in annexin V assay (Figure 4A,B), both napabucasin and 2′-methyl napabucasin gave the total apoptotic cells ~40%. After 48 h, the napabucasin series significantly induced the apoptotic mechanisms on TF1 cells similar to both drugs, tofacitinib and ruxolitinib. Interestingly, a low level of apoptosis was found after 24-h treatment with compounds and drugs. All compounds gave a high level of apoptotic cells after 72 h treated. In addition, the number of apoptotic cells treated by napabucasin series and drugs from the caspase 3/7 assay method was dramatically increased after 24, 48, and 72 h incubation, respectively (Figure 5A,B). Altogether, the annexin V and caspase 3/7 results of the napabucasin series and drugs treated displayed similar phenomena by giving a high level of apoptotic cells. 

Furthermore, the dose-dependent effects in the napabucasin series and drug treatment were examined with various concentrations at IC_25_, IC_50_, and IC_75_ values for 24 h and analyzed by annexin V assay (Figure S5). Noticeably, after 24 h at IC_25_ value treatment, the napabucasin series and drugs showed a low level of apoptotic cells (20 to 30%). High levels of apoptotic cells (~40%) were observed after treatment with napabucasin series and drugs at IC_75_ value. These findings indicated that napabucasin and 2′-methyl napabucasin significantly induced the cytotoxicity via apoptosis in TF1 cells in a time and dose-dependent manner. Corresponding with other quinone analogs, emodin and butoxy mansonone G induced apoptosis in the RPMI8226 myeloma, A549, and H1975 lung cancer cell lines [37,38]. In addition, the naphtoquinone–coumarin conjugate can also inhibit myelogenous leukemia K562 cells by apoptosis in a time (24 and 48 h) and does-dependent (5 and 10 μM) [33] manners, while naphthoquinone–benzonitrile conjugate induced cell death via apoptosis in a time-dependent manner (24, 48 and 72 h) [14]. 

### 2.4. Binding Pattern of Napabucasin Series with JAK2/3 by Molecular Docking

**Figure 6:**
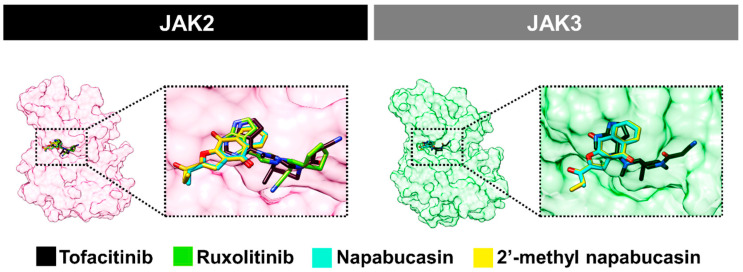
Superimposition of napabucasin, 2′-methyl napabucasin, tofacitinib, and ruxolitinib within JAK2/3 derived from GOLD docking.

Because the napabucasin and 2′-methyl napabucasin gave potent JAK2/3 inhibition and also inhibited TF1 and HEL cell growth, the binding patterns of both compounds in comparison with drugs, tofacitinib and ruxolitinib, were investigated using molecular docking (Figure 6). The orientation of napabucasin and 2′-methyl napabucasin within JAK2/3 displayed similar phenomena. In the JAK2 system, the naphthoquinone core structure of both napabucasin series demonstrated a similar pattern. Furthermore, the core of naphthoquinone overlapped with the pyrrolopyrimidine of both drugs, tofacitinib and ruxolitinib. Likewise, in the JAK3 system, the naphthoquinone motif of both napabucasin analogs aligned similarly to tofacitinib. A similar orientation of napabucasin series within JAK2 and JAK3 has been found. Therefore, the only system of the JAK2/napabucasin series was selected to study the binding affinity by MD simulations. 

### 2.5. System Stability of JAK2 with Napabucasin Series

**Figure 7:**
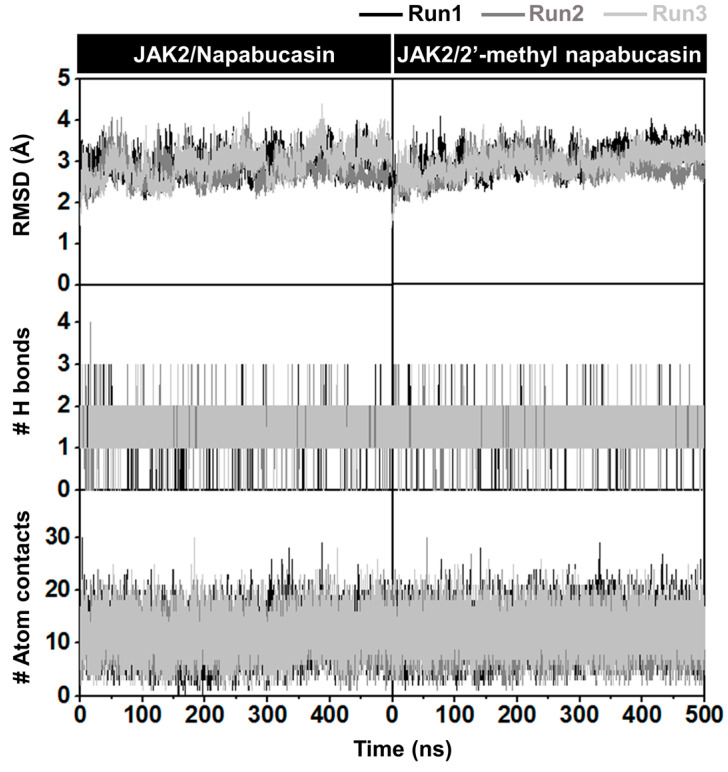
All-atom RMSD, #H-bonds, and #atom contacts of complexes between JAK2 and napabucasin series along 500 ns simulations.

**Figure 8:**
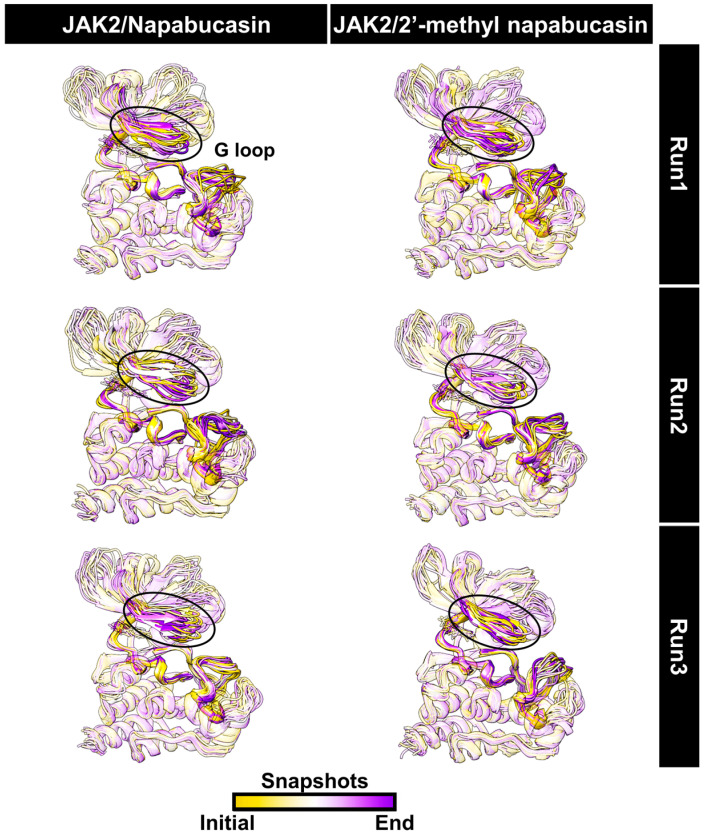
Snapshot per time (25 frames) of complexes between JAK2 and napabucasin series along 500 ns simulations.

The complexes between JAK2 and both napabucasin series were investigated at 500 ns simulations in terms of the root-mean-square deviation (RMSD) of atomic positions, the number of hydrogen bonding (#H-bonds), and the number of atom contacts (#atom contacts), as illustrated in Figure 7. The RMSD values of complexes between napabucasin and 2′-methyl napabucasin with JAK2 slightly increased at the first 100 ns and stably maintained at a fluctuation of ~2.0−3.5 Å until the end of simulation time. Moreover, the #H-bonds number (~1–3) and #atom contacts (~11–12) of both systems were quite stable along with the simulation. In addition, the MD snapshot per time from three independent simulations of napabucasin and 2′-methyl napabucasin complexes with JAK2 was obtained (Figure 8), compared with JAK2/ruxolitinib system [39] (Figure S6). The G loop of each napabucasin and 2′-methyl napabucasin complex showed slight fluctuation in the pocket site. It implies that both napabucasin series could bind well in the JAK2 binding pocket. Altogether, the last 100 ns MD trajectories of each system were selected for further study. 

### 2.6. Binding Patterns of JAK2 with Napabucasin Series

**Figure 9:**
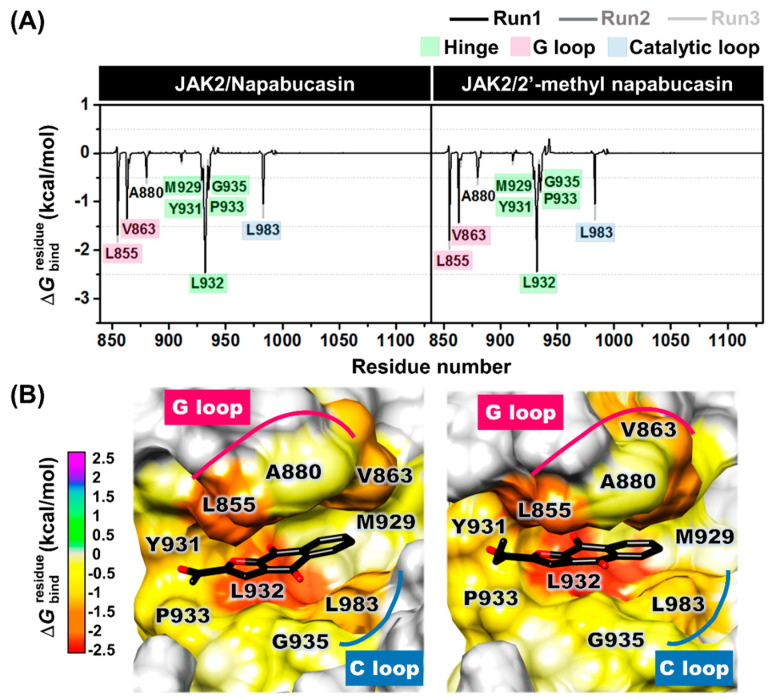
(**A**) Decomposition free energy ($\Delta G_{bind}^{residue}$) of complexes between JAK2 and napabucasin series and (**B**) their average binding pattern of napabucasin series within JAK2 in ATP binding pocket from the average three independent simulations (last 100 ns). The lowest and highest energies ranged from red to magenta, respectively.

The binding patterns of JAK2 with napabucasin series, napabucasin, and 2′-methyl napabucasin were investigated using the MM/GBSA ($\Delta G_{bind}^{residue}$) calculation derived from the last 100 ns (100 snapshots) in the three independent simulations. The compound’s orientation within the JAK2 pocket site is displayed in Figure 9. The $\Delta G_{bind}^{residue}$ in negative and positive values represents stabilization and destabilization, respectively. The energy per residue of both napabucasin and 2′-methyl napabucasin with JAK2 from each run showed a similar pattern. 

The hydrophobic residues that exhibited an energy contribution of <−0.5 kcal/mol were considered in Figure 9A. Napabucasin and 2′-methyl napabucasin were strongly stabilized similarly with residues, as follows (Figure 9B): (i) L855 (dark orange) and V863 (orange) at G loop, (ii) M929 (light yellow), Y931 (dark yellow), L932 (red), P933 (yellow) and G935 (yellow) at the hinge region, (iii) L983 (light orange) located at the catalytic loop (C loop) and another region, and near G loop was A880 (yellow). In addition, the methyl group of napabucasin and ethyl group of 2′-methyl napabucasin pointed into the P933 at the hinge region were found. These findings corresponded well with hydrophobic regions, for example, pyrrolopyrimidine and piperidine rings of tofacitinib can tightly interact with L855, V863, A880, Y931, L932, and L983 of JAK2, as previously reported [40]. In addition, previous reports revealed that L855, V863, A880, and L983 of JAK2 showed hydrophobic interactions with pyrrolopyrimidine and pyrazolone rings of ruxolitinib [39,41], the aromatic ring of the naphthoquinone-coumarin conjugate [33] and quinazoline template of compound **5k** (4-methylmorpholine derivative) [42], which agree with this work. Therefore, it was indicated that the hydrophobic interaction plays a vital role in binding between JAK2 and drugs, as well as the napabucasin series. 

**Figure 10:**
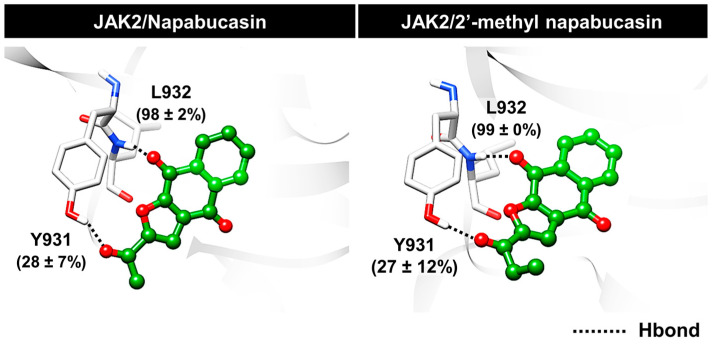
Hydrogen bond occupation of napabucasin series within JAK2 from the average three independent simulations (last 100 ns).

Furthermore, hydrogen bonding, one of the binding strengths of ligand binding, was investigated. The napabucasin (98 ± 2%) and 2′-methyl napabucasin (99 ± 0%) were strongly stabilized with L932 at hinge regions via hydrogen bonds. In addition, in this region, the hydrogen bonds were still found with Y931 with the 28 ± 7% occupation for napabucasin and 27 ± 12% for 2′-methyl napabucasin (Figure 10). This finding is in agreement with previous studies showing that L932 residues of JAK2 displayed essential binding via hydrogen bonds between pyrrolopyrimidine core structure of drugs, tofacitinib [40] and ruxolitinib [41], as well as naphthoquinone-coumarin conjugate [33] at the ATP-binding site. As illustrated in Figure S7, the energy contribution revealed that the complexes between JAK2 and napabucasin, as well as 2′-methyl napabucasin were stabilized by van der Waals contribution (Δ*E*_vdw_ + Δ*G*_solv, nonpolar_) rather than electrostatic contribution (Δ*E*_elec_ + Δ*G*_solv, polar_), similar to previous research of thienopyridine binding within JAK2 [43]. 

## 3. Materials and Methods

### 3.1. Preparation of Naphthoquinone Analogs

Juglone, menadione, and lawsone were purchased from Sigma-Aldrich with purity ≥97%. Napabucacin and 2′-methyl napabucasin, which are classified as furanonaphthoquinone, were synthesized based on the reported procedure [26]. 

*Synthesis of napabucacin*. To a stirred solution of methyl vinyl ketone (2.5 equiv, 7.18 mmol. 0.61 mL) in pentane (6.0 mL) was drop-wisely added a solution of bromine (2.6 equiv, 7.46 mmol, 0.30 mL) in pentane (3.0 mL) at −15 °C. The reaction was continuously stirred for 10 min. After that, all volatile substances were removed under reduced pressure to provide the crude 1,2-dibromobutan-2-one. Next, 2-hydroxy-1,4-naphthoquinone or lawsone (1 equiv, 2.87 mmol, 500 mg) was dissolved in dry tetrahydrofuran (THF, 15 mL). The reaction mixture was added 1,8-diazabicyclo(5.4.0)undec-7-ene (DBU, 3.7 equiv, 10.62 mmol, 1.60 mL), resulting in the red solution. The reaction was stirred at 0 °C for 20 min under the nitrogen atmosphere. Then, the solution of crude 1,2-dibromobutane in dry THF (3 mL) was added. After addition, the reaction mixture was gradually warmed to room temperature for 30 min, followed by refluxing for 5 h. To the reaction was added another portion of DBU (0.52 equiv, 1.49 mmol, 0.22 mL) and was continuously refluxed for the additional 3 h. The reaction was quenched with a saturated ammonium chloride-water solution and extracted with chloroform (25 mL, 3 times). The organic layers were combined, washed with water, dried over anhydrous sodium sulfate (NaSO_4_), filtered, and concentrated under reduced pressure to obtain a crude product. Purification of the crude product by flash column chromatography using the gradient hexane and dichloromethane solutions as the eluting solvents. Napabucacin was obtained as a yellow solid at 420 mg (61%). ^1^H-NMR (CDCl_3_, 300 MHz) δ 8.26 (1H, m, 5-H), 8.25 (1H, m, 8-H), 7.81 (1H, m, 6-H), 7.81 (1H, m, 7-H), 7.61 (1H, s, 3-H), 2.67 (3H, s, 11-H); ^13^C-NMR (CDCl_3_, 75 MHz) δ 187.5 (C-10), 179.7 (C-4), 173.9 (C-9), 155.5 (C-9a), 152.9 (C-2), 134.4 (C-6), 134.3 (C-7), 133.1 (C-8a), 132.6 (C-4a), 130.7 (C-3a), 127.4 (C-8), 127.2 (C-5), 112.3 (C-3), 26.8 (C-11); IR (KBr) 3113, 3014, 2854, 1690, 1674, 1581, 1359, 1285, 1259, 1224, 1197, 977, 875, 717 cm^−1^; HRMS-ESI m/z 263.0321 ([M+Na]^+^, calcd for C_14_H_8_O_4_Na^+^ 263.0315) (Figures S8 and S9).

*Synthesis of 2′-methyl napabucasin*. To prepare the propionyl-containing furanonaphthoquinone, pen-1-en-3-one was employed and subjected to the same protocol as the synthesis of napabucasin yielding 2′-methyl napabucasin at 426 mg (56%) as a yellow solid. ^1^H-NMR (CDCl_3_, 300 MHz) δ 8.26 (1H, m, 5-H), 8.23 (1H, m, 8-H), 7.81 (1H, m, 6-H), 7.81 (1H, m, 7-H), 7.61 (1H, s, 3-H), 3.06 (2H, q, *J* = 7.5, 14.7 Hz, 11-H), 1.26 (3H, t, *J* = 7.2 Hz, 12-H); ^13^C-NMR (CDCl_3_, 75 MHz) δ 190.8 (C-10), 179.8 (C-4), 173.9 (C-9), 155.4 (C-9a), 152.7 (C-2), 134.4 (C-7), 134.3 (C-6), 133.1 (C-8a), 132.6 (C-4a), 130.7 (C-3a), 127.3 (C-5), 127.3 (C-8), 112.0 (C-3), 32.6 (C-11), 7.4 (C-12); IR (KBr) 3385, 3115, 2978, 2878, 1698, 1672, 1579, 1567, 1354, 1219, 958, 721 cm^−1^; HRMS-ESI m/z 277.0464 ([M+Na]^+^, calcd for C_15_H_10_O_4_Na^+^ 277.0471) (Figures S10 and S11).

### 3.2. Cell Culture and Cytotoxicity of Leukemia Cell Lines

The human erythroleukemia TF1 (ATCC CRL-2003) and HEL 92.1.7 (ATCC TIB-180) cells, which purchase from ATCC were grown in complete RPMI-1640 medium supplemented with fetal bovine serum (FBS, 10% *v*/*v*), 100 U/mL penicillin, and 100 μg/mL streptomycin. To the complete RPMI-1640 medium for TF1 cells was added 2 ng/mL of granulocyte-macrophage colony-stimulating factor (GM-CSF). Both cells were maintained at 37 °C in a 5% (*v*/*v*) CO_2_, 95% (*v*/*v*) air-humidified incubator.

The cytotoxicity activity of naphthoquinones against both TF1 and HEL cells was performed using the Presto Blue assay. The TF1 (50,000 cells/well) and HEL (25,000 cells/well) cell suspensions were seeded in a 96-well microplate and incubated overnight at 37 °C. Then, both cells were treated at 10 μM of compounds and known drugs (tofacitinib and ruxolitinib) and were incubated for 72 h. The absorbance of the resorufin product was measured at 570 nm using a microplate reader (Infinite M200 microplate reader, Tecan, Männedorf, Switzerland) after adding 10 μL of Presto Blue reagent in TF1 and HEL cells, which incubated at 37 °C for 1 h. The treatment without compounds/known drugs was used as vehicle control. 

### 3.3. Kinase Inhibitory Activity of JAK2/3

The tyrosine kinase inhibition of JAK2 and JAK3 was performed using the ADP-Glo™ kinase assay (Promega). The 2.5 ng/μL of enzymes (Sigma-Aldrich), JAK2 (SRP0171), and JAK3 (SRP0173) were mixed with 1 μM of compounds/known drugs, 5 μM ATP and 2 ng/μL poly(glu·tyr), in a buffer (40 mM Tris−HCI pH 7.5, 20 mM MgCl_2_, and 0.1 mg/mL BSA). The reactions were incubated for 1 h at room temperature. Then, 5 μL of the ADP-Glo reagent was added and incubated for 40 min. Next, 10 μL of kinase detection reagent was added and incubated at room temperature for 30 min to convert ADP to ATP product. The ATP was detected by measuring the luminescence using a microplate reader. The relative inhibition (%) of compounds/known drugs was calculated and compared to the control without compounds/known drugs. 

### 3.4. Apoptosis Detection Assay

After treatment, TF1 cells followed by time (24, 48, and 72 h) and dose-dependent (IC_25_, IC_50,_ and IC_75_ at 24 h) of compounds/known, the treated cells were gently trypsinized for 5 min. The cells were then harvested after being centrifuged at 1000 rpm for 5 min. Then, the pellets were resuspended and incubated for 25 min at room temperature in the dark with annexin V and dead cell reagent (MCH100105, Merk, Germany). The apoptosis was assessed by a flow cytometry MUSE (Merck-MiIIipore, Burlington, MA, USA) analyzer. All experiments were independently performed three times. 

### 3.5. Caspase Assays

After TF1 cells were treated with an IC_50_ value of compounds/known at 24, 48, and 72 h, the cells were gently trypsinized for 5 min. Subsequently, cells were harvested after being centrifuged at 1000 rpm for 5 min. The pellets were resuspended and incubated with 5 μL of caspase 3/7 reagent (MCH100108, Merk, Germany). Reactions were then incubated at 37 °C for 30 min and then mixed with 7-aminoactinomycin D (7-ADD). The number of apoptosis cells was analyzed by flow cytometry using the cell analyzer MUSE (Merck-MiIIipore, Burlington, MA, USA). All experiments were independently performed three times. 

### 3.6. Statistical Analysis

The data from the triplicate independent experiment are represented as mean ± standard error of the mean (SEM) using GraphPad Prism 7.0 software (GraphPad Prism Software Inc., San Diego, CA, USA). Data were statistically evaluated using one-way and two-way analysis of variance (ANOVA) in which Tukey’s multiple comparisons with confidence level *p* ≤ 0.05.

### 3.7. Molecular Docking

The three-dimensional X-ray structures of tofacitinib complexes with JAK2 (code 3FUP) and JAK3 (code 3LXK) were downloaded from Protein Data Bank [10,44]. All missing residues (920–923) of JAK2 were built using the SWISS-MODEL server [45]. The protonation states were predicted by ionizable amino acids at physiological pH of 7.4 using PROPKA 3.1 [46]. The three-dimensional structures of naphthoquinones were generated and characterized by the Gaussian09 program [47] and ChemAxon [48], respectively. The ligand was optimized using the Gaussian09 program at the level of HF/6–31d as the previous protocol [40]. 

Tofacitinib was defined as the center of the active site in both JAKs for GOLD docking programs, which are genetic algorithms (GA) [49,50]. The docking parameter was12 Å of sphere radius, 100 independent docking pose, GOLD score, and ChemScore (rescore) for scoring function. The docking results were visualized for binding patterns by the UCSF Chimera package [51] and Accelrys Discovery Studio 2019 (Accelrys Inc., San Diego, CA, USA) [52].

### 3.8. Molecular Dynamics Simulations 

The three different initial velocities of all-atom MD simulations of the JAK2 complex with naphthoquinones were simulated under explicit water solvation (TIP3P model) using AMBER16 as in previous studies [53,54,55]. The periodic boundary condition with the isothermal–isobaric ensemble (NPT) at a constant pressure of 1 atm equilibrated at 310 K was used. The JAK2 and naphthoquinones force fields were FF14SB [56] and GAFF2 [57], respectively. The electrostatic potential (ESP) charges and restrained ESP (RESP) were adopted in AMBER16. For non-bonded interactions were used for the cutoff of 12 Å, while long-range electrostatic interactions were applied by Ewald’s method [58]. The SHAKE algorithm was used to constrain all covalent bonds involving hydrogen atoms [59]. The temperature was controlled by a Langevin thermostat with a collision frequency of 2.0 ps. The NPT ensemble was simulated for 500 ns, which recorded the MD trajectories every 500 steps. The system analysis was performed by the root mean square displacement (RMSD), intermolecular hydrogen bonds occupation, and atom contacts using the CPPTRAJ module [60]. Decomposition free energy was calculated by the MM/PBSA.py module. 

## 4. Conclusions

The search for naphthoquinones that could inhibit JAK2/3 was carried out using a combination of experimental and computational strategies. We found the napabucasin and 2′-methyl napabucasin for JAK2/3 inhibitions. Both compounds strongly inhibited JAK2/3 in a nanomolar range in the kinase experiment. Similar to the known drugs tofacitinib and ruxolitinib, the napabucasin and 2′-methyl napabucasin demonstrated inhibition of TF1 (IC_50_ = 9.57 and 18.10 μM) and HEL (IC_50_ = 3.31 and 6.65 μM) cell growths. These compounds have a time- and dose-dependent ability to cause TF1 cell death to apoptosis. According to the molecular dynamics analysis, both compounds stabilized within the pocket by hydrophobic contacts with conserved regions, hinge region, G loop, and catalytic loop of JAK2 and formed hydrogen bonds with the Y931 and L932 residues. These findings imply that it will be possible to develop potentially effective targeted small molecule JAK2/3 inhibitors. 

## Figures and Tables

**Table 1:** The IC_50_ value of JAK2/3 inhibitions of napabucasin series and known drugs, tofacitinib and ruxolitinib.

Compounds	IC_50_ ± SEM (nM)	
	JAK2	JAK3
Napabucasin	12.62 ± 2.12 ***	14.84 ± 2.37
2′-methyl napabucasin	11.11 ± 0.13 ***	10.24 ± 0.66 **
Tofacitinib	32.10 ± 1.99	21.03 ± 0.97
Ruxolitinib	14.63 ± 0.81	nd

** *p* ≤ 0.01 and *** *p* ≤ 0.001 compared with tofacitinib, nd refers to not detected.

## Data Availability

Not applicable.

## Supplementary Materials

The following supporting information can be downloaded at: https://www.mdpi.com/article/10.3390/molecules28020597/s1. Figure S1: Cytotoxicity of naphthoquinones at 10 μM against TF1 and HEL cells.; Figure S2: IC_50_ for cell viability inhibition of naphthoquinones and known drugs (tofacitinib and ruxolitinib) in TF1 cells.; Figure S3: IC_50_ for cell viability inhibition of naphthoquinones and known drugs (tofacitinib and ruxolitinib) in HEL cells.; Figure S4: IC_50_ for JAK2/3 inhibitions of napabucasin and 2′-methyl napabucasin comparisons with known drugs, tofacitinib and ruxolitinib.; Figure S5: Dose-dependent effect of napabucasin and 2′-methyl napabucasin comparison with drugs, tofacitinib and ruxolitinib at IC_25_, IC_50_ and IC_75_ value on the apoptosis of TF1 cells by annexin V for 24 h.; Figure S6: Snapshot per time (25 frames) of complex between JAK2 and ruxolitinib along 500 ns simulations.; Figure S7: Average energy contribution between napabucasin series within JAK2 in ATP binding pocket from the average three independent simulations (last 100 ns). Figures S8–S11: ^1^H and ^13^C NMR Spectra of napabucasin and 2′-methyl napabucasin.

## Sample Availability

Samples of the compounds are available from the authors.
