# Supplementary material for: In Silico and In Vitro Study of Janus Kinases Inhibitors from Naphthoquinones
Source: Molecules. 2023 Jan 6;28(2):597. doi: 10.3390/molecules28020597 (PMC9866339; doi:10.3390/molecules28020597)
Supplement: Supplementary file 1 [file molecules-28-00597-s001.zip › molecules-2098114-supplementary.pdf]

Supporting Information

In silico and *in vitro* study of Janus kinases inhibitors from naphthoquinones

Kamonpan Sanachai[1], Panupong Mahalapbutr[2], Lueacha Tabtimmai[3], Supaphorn Seetaha[4], Nantawat Kaekratoke[5], Supakarn Chamni[6,7], Syed Sikander Azam[8], Kiattawee Choowongkomon[4,*], Thanyada Rungrotmongkol[1,9,*]

[1] Center of Excellence in Biocatalyst and Sustainable Biotechnology, Department of Biochemistry, Faculty of Science, Chulalongkorn University, Bangkok 10330, Thailand

[2] Department of Biochemistry, Faculty of Medicine, Khon Kaen University, Khon Kaen 40002, Thailand

[3] Department of Biotechnology, Faculty of Applied Science, King Mongkut's University of Technology of North Bangkok, Bangkok 10800, Thailand

[4] Department of Biochemistry, Faculty of Science, Kasetsart University, Bangkok 10900, Thailand

[5] Department of Materials Science and Engineering, Vidyasirimedhi Institute of Science and Technology (VISTEC), Rayong, 21210, Thailand

[6] Department of Pharmacognosy and Pharmaceutical Botany, Faculty of Pharmaceutical Sciences, Chulalongkorn University, Bangkok 10330, Thailand

[7] Natural products and Nanoparticles Research Unit (NP2), Faculty of Pharmaceutical Sciences, Chulalongkorn University, Bangkok 10330, Thailand

[8] Computational Biology Lab, National Center for Bioinformatics, Quaid-i-Azam University, Islamabad, Pakistan

[9] Program in Bioinformatics and Computational Biology, Graduate School, Chulalongkorn University, Bangkok 10330, Thailand

* Correspondences: t.rungrotmongkol@gmail.com (T.R.) and fsciktc@ku.ac.th (K.C.); Tel.: +66 2 2185426 (T.R.)

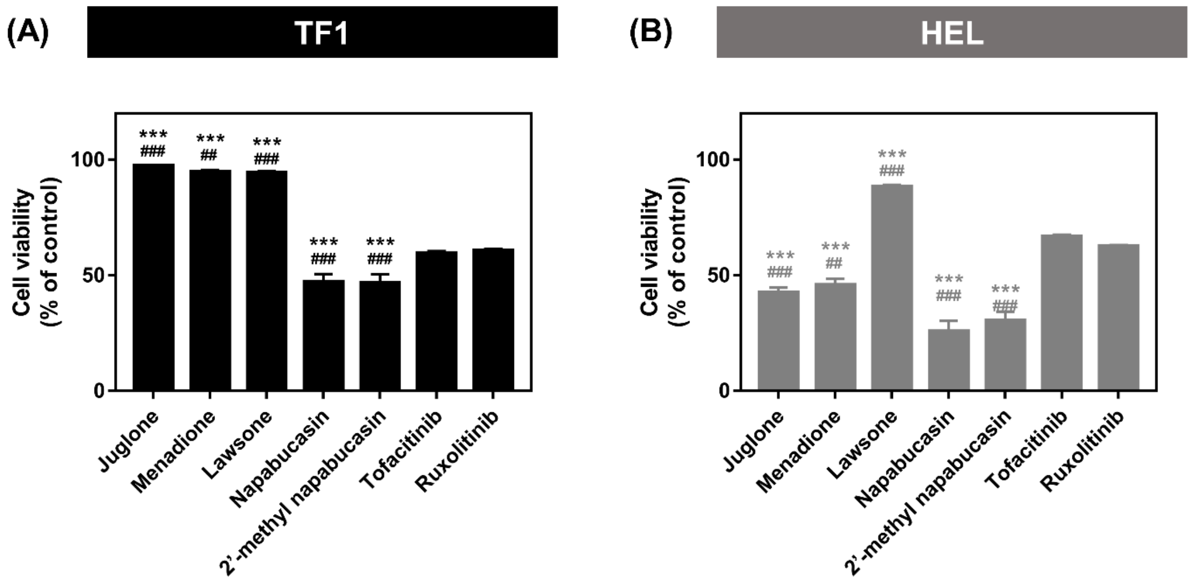

Figure S1. Cytotoxicity of naphthoquinones at 10 μM against TF1 and HEL cells by Presto Blue assay. *** $p \leq 0.001$ vs. tofacitinib, ## $p \leq 0.01$ and ### $p \leq 0.001$ vs. ruxolitinib.

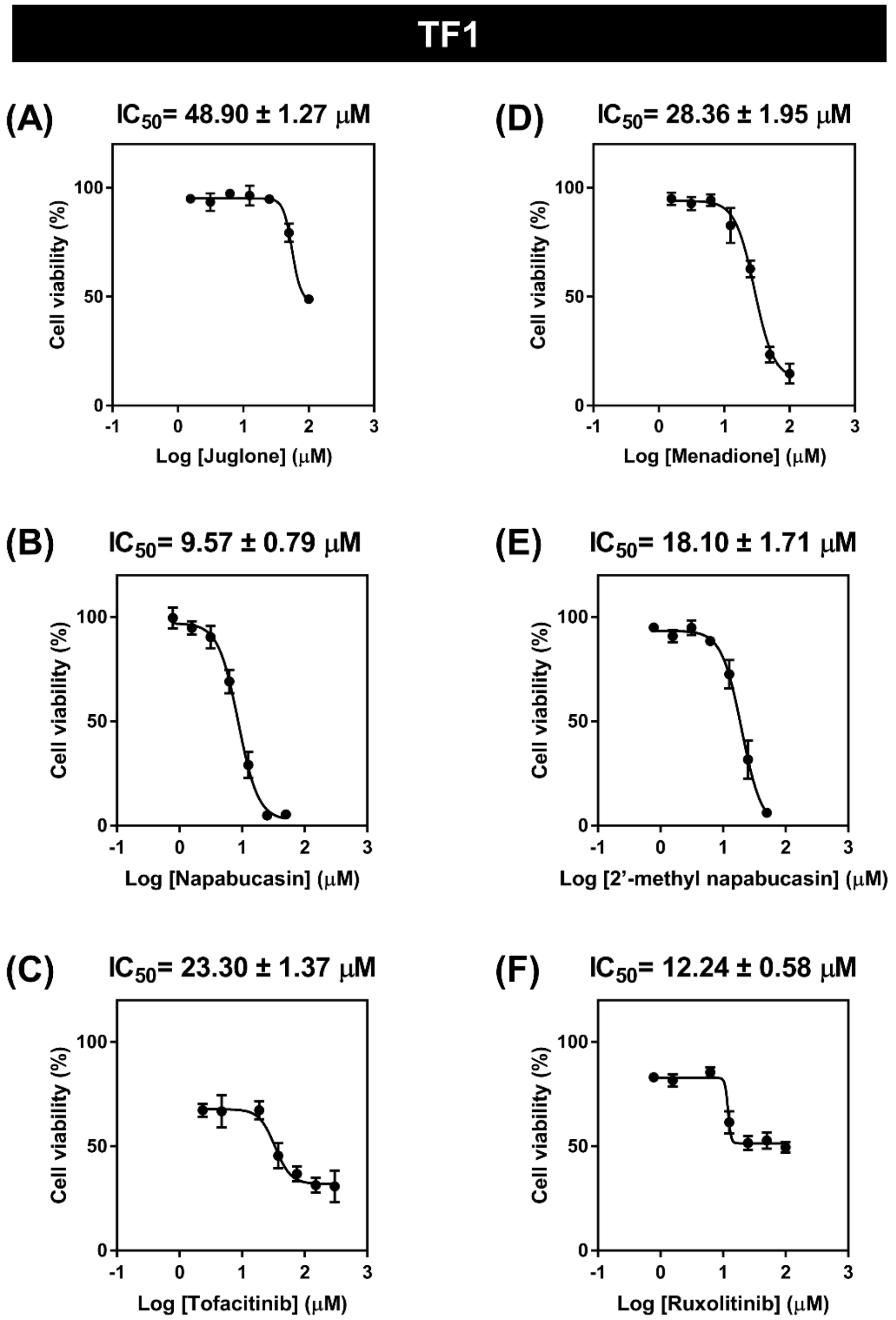

Figure S2. IC_{50} for cell viability inhibition of naphthoquinones and known drugs (tofacitinib and ruxolitinib) in TF1 cells by Presto Blue assay.

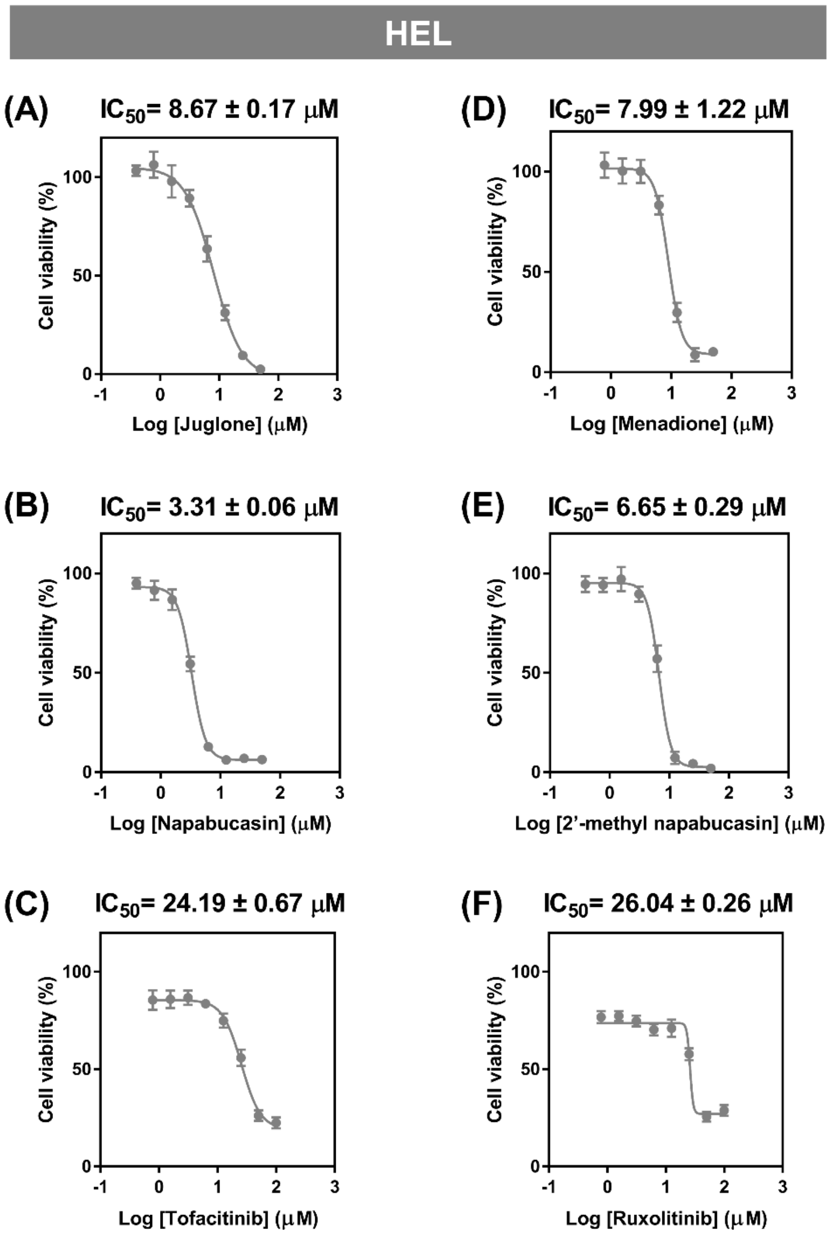

Figure S3. IC_{50} for cell viability inhibition of naphthoquinones and known drugs (tofacitinib and ruxolitinib) in HEL cells by Presto Blue assay.

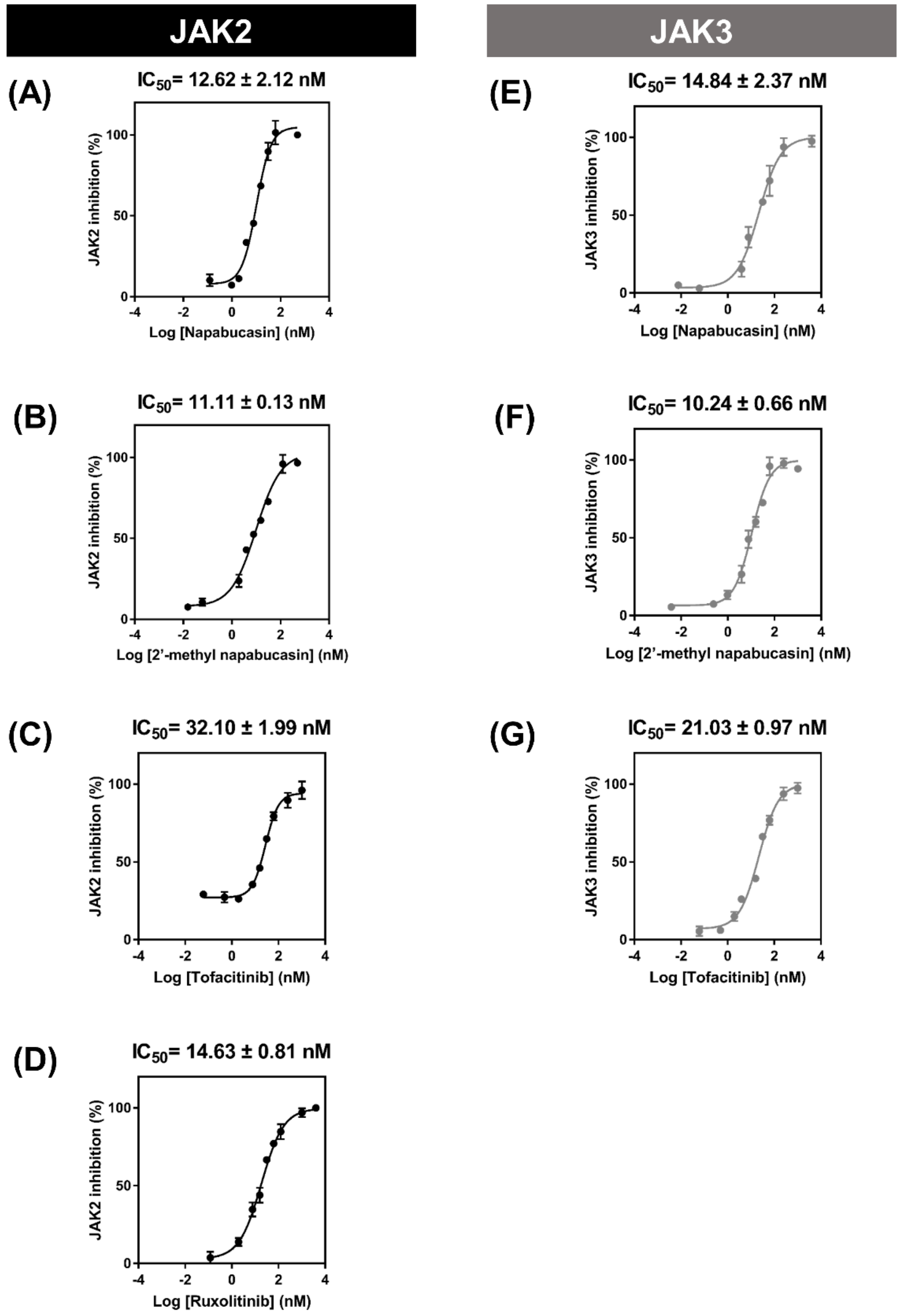

Figure S4. IC_{50} for JAK2/3 inhibitions of napabucasin and 2′-methyl napabucasin comparisons with known drugs, tofacitinib and ruxolitinib by ADP-Glo™ kinase assay.

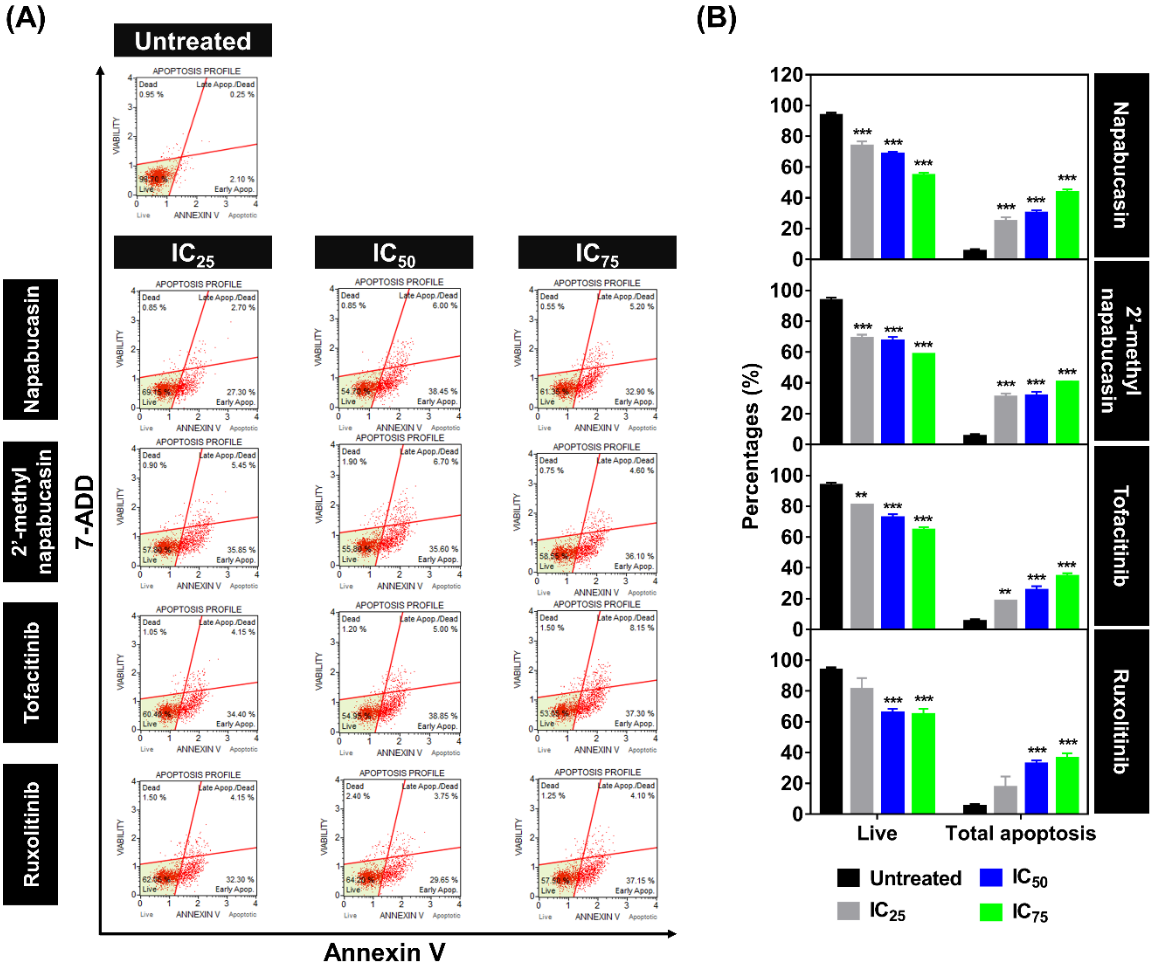

Figure S5. Dose-dependent effect of napabucasin and 2'-methyl napabucasin comparison with drugs, tofacitinib and ruxolitinib at IC_{25}, IC_{50} and IC_{75} values (nM) on the apoptosis of TF1 cells by annexin V for 24 h. (A) The flow cytometry dot plot of each group which divided into four quadrants: live cells, early apoptotic cells, late apoptotic cells, and necrotic cells. (B) The percentages of live cells and apoptotic cells. **p* ≤ 0.05, ***p* ≤ 0.01 and ****p* ≤ 0.001 compared with the untreated group.

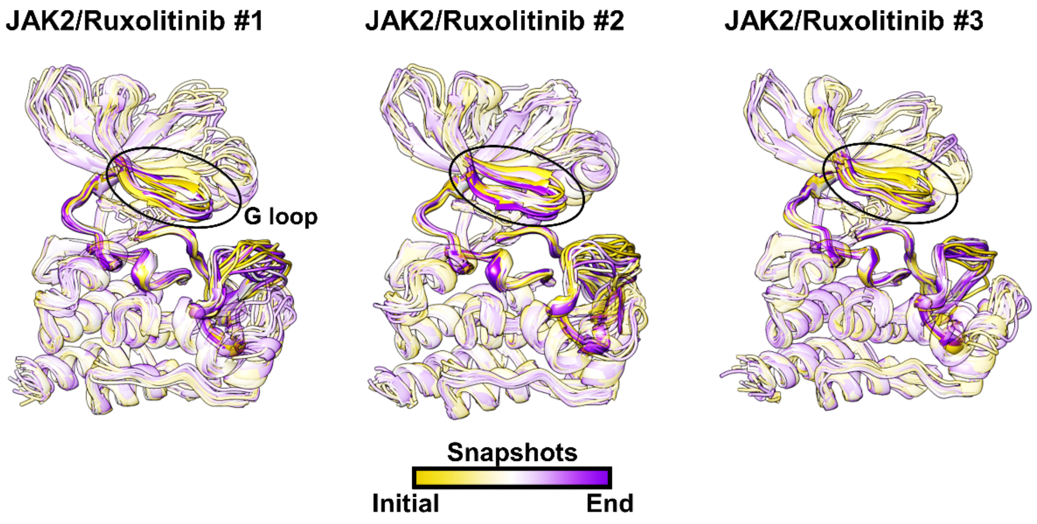

Figure S6. Snapshot per time (25 frames) of complex between JAK2 and ruxolitinib along 500 ns simulations.

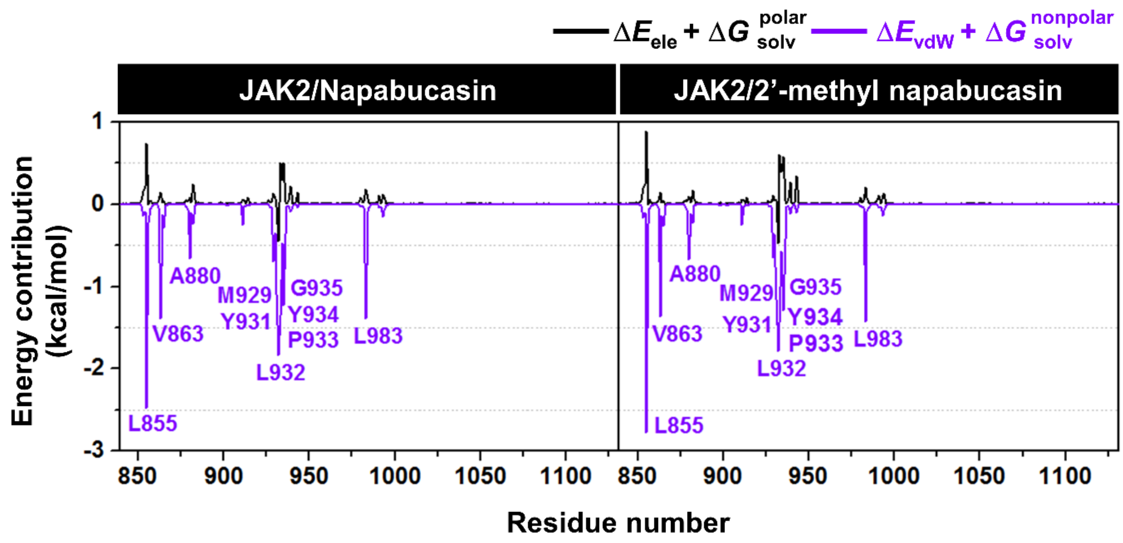

Figure S7. Energy contribution between napabucasin series within JAK2 in ATP binding pocket from the average three independent simulations (last 100 ns).

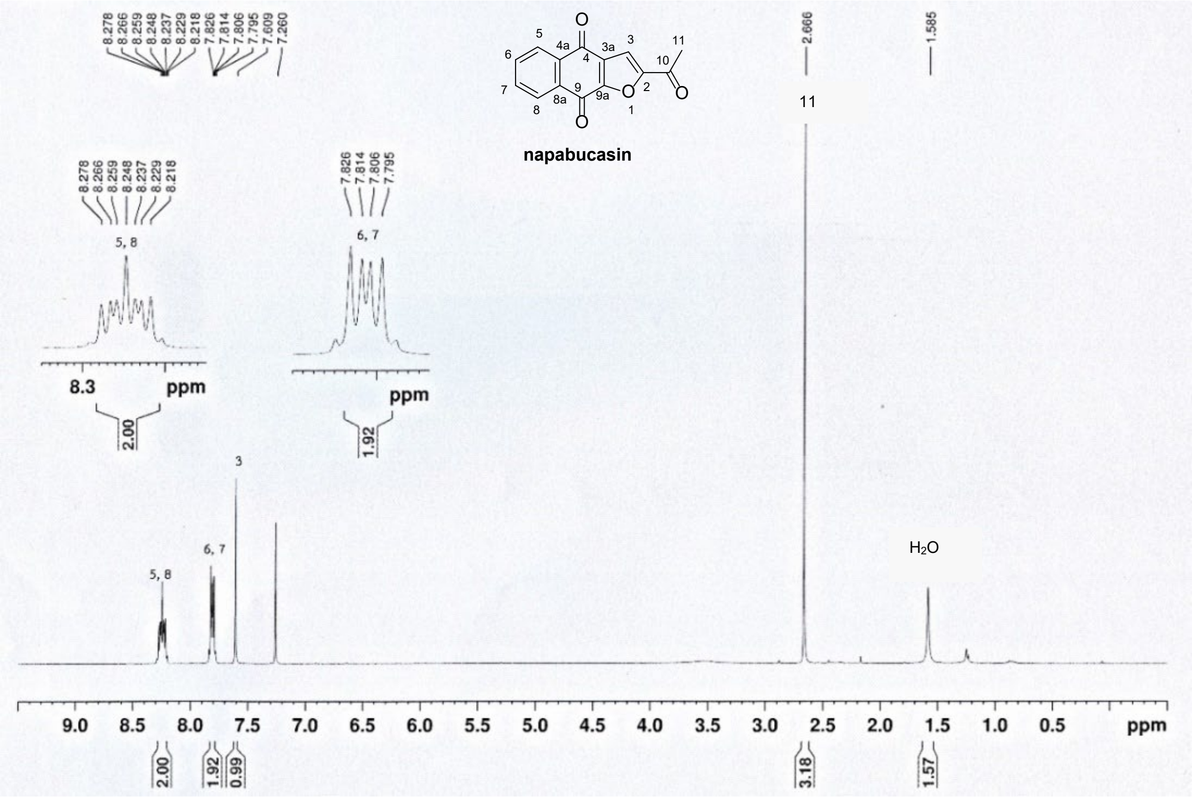

Figure S8. ^{1}H NMR (300 MHz) spectrum of napabucasin in $CDCl_3$.

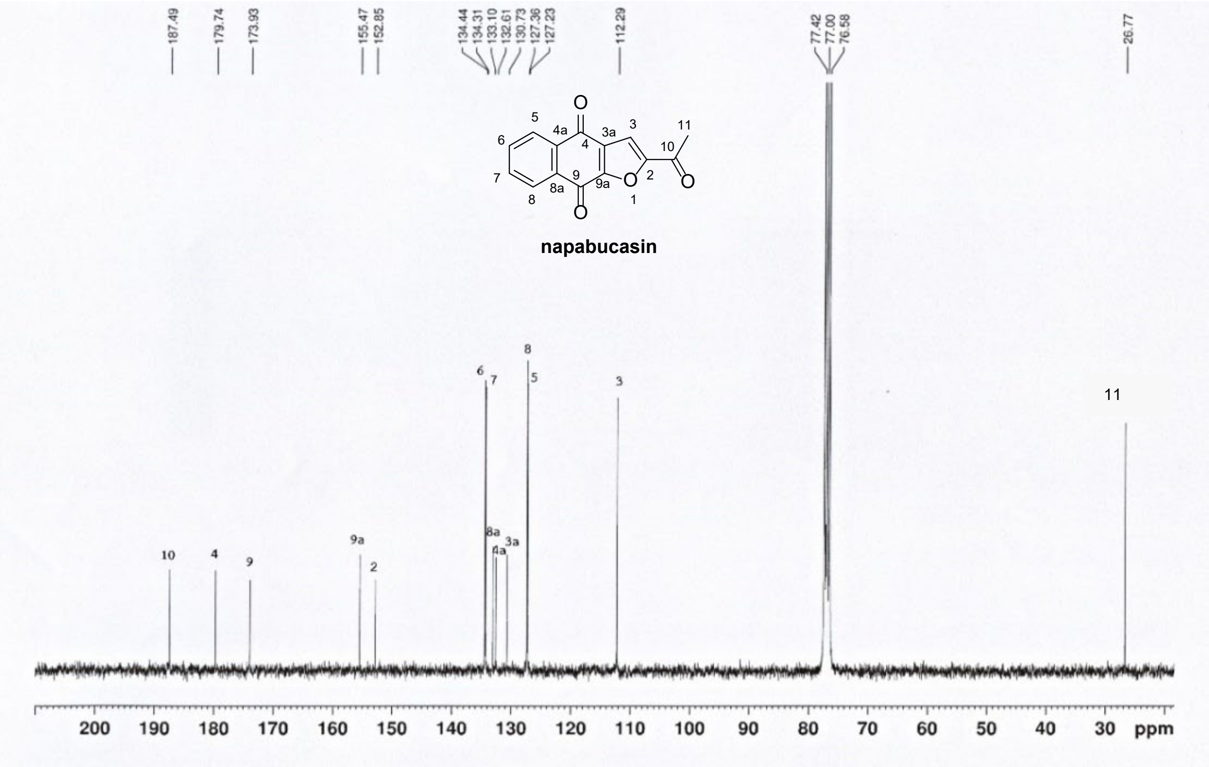

Figure S9. ^{13}C NMR (100 MHz) spectrum of napabucasin in $CDCl_3$.

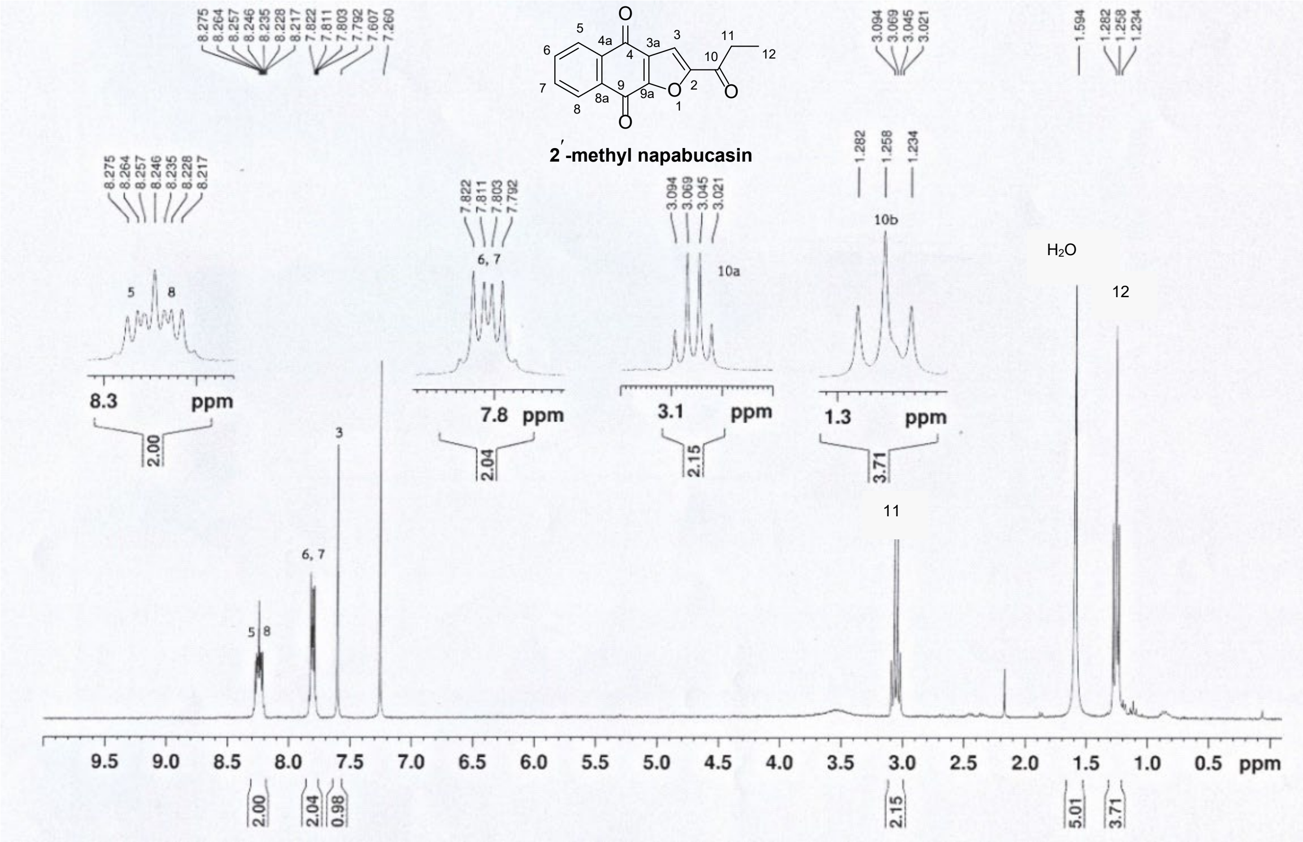

Figure S10. ^{1}H NMR (300 MHz) spectrum of 2′-methyl napabucasin in $CDCl_3$.

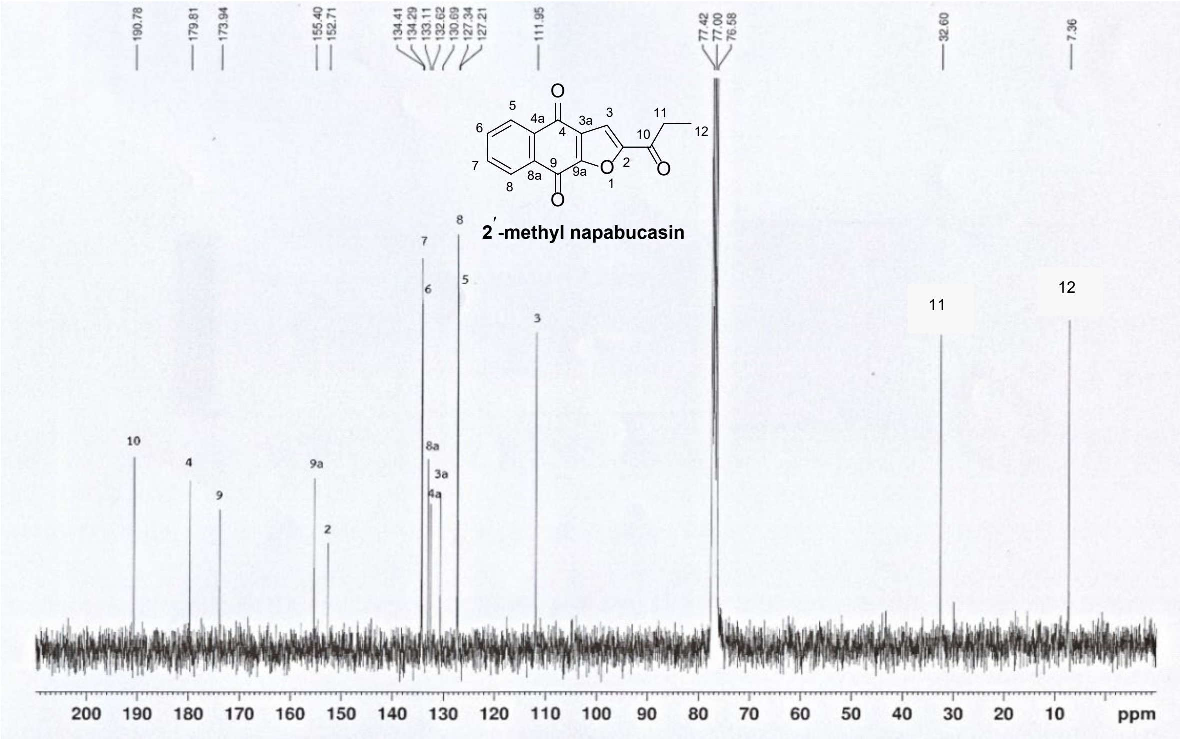

Figure S11. ^{13}C NMR (100 MHz) spectrum of 2′-methyl napabucasin in $CDCl_3$.
